# *In-silico* tool for predicting and scanning rheumatoid arthritis-inducing peptides in an antigen

**DOI:** 10.3389/fimmu.2025.1630863

**Published:** 2025-09-01

**Authors:** Ritu Tomer, Shipra Jain, Pushpendra Singh Gahlot, Nisha Bajiya, Gajendra P. S. Raghava

**Affiliations:** Department of Computational Biology, Indraprastha Institute of Information Technology, New Delhi, India

**Keywords:** autoimmune disease, rheumatoid arthritis, T-cell epitopes, machine learning, large language models

## Abstract

**Introduction:**

Rheumatoid arthritis (RA) is an autoimmune disorder in which the immune system mounts an abnormal response to self-antigens, resulting in chronic inflammation and joint damage. Identifying antigenic regions in proteins that trigger RA is essential for the development of protein-based therapeutics.

**Methods:**

We developed predictive models for HLA class II binding RA-inducing peptides using a dataset of 291 experimentally validated RA-inducing peptides and 165 RA non-inducing peptides. Positional and compositional analyses were performed to identify residue preferences. Alignment-based approaches (BLAST and MERCI), machine learning classifiers, deep learning, and protein language model–based methods were evaluated for predictive performance.

**Results:**

Compositional analysis revealed significant enrichment of glycine, proline, and tyrosine in RA-inducing peptides. Alignment-based approaches provided high precision but limited coverage. Among machine learning methods, XGBoost achieved the best performance (AUC = 0.75) on the validation dataset, while ProtBERT was the top-performing protein language model (AUC = 0.72). The ensemble model integrating XGBoost with MERCI-derived motifs yielded the highest overall performance (AUC = 0.80; MCC = 0.45) on an independent validation dataset.

**Discussion:**

This study presents computational strategies for identifying RA-inducing peptides and demonstrates the advantage of combining motif-based and machine learning approaches for improved performance. The findings are valuable for evaluating the safety of proteins in probiotics, genetically modified foods, and protein-based therapeutics. To facilitate broader use, the best-performing approach has been implemented in RAIpred, a web server and standalone software tool for predicting and scanning RA-inducing peptides, available at https://webs.iiitd.edu.in/raghava/raipred/.

## Highlights

- Rheumatoid arthritis (RA), an incurable chronic joint disorder.
- Identification of antigenic regions responsible for inducing RA.
- Application of protein language models in prediction of RA-inducing peptides.
- Ensemble model integrating similarity-based and machine learning approaches.
- Development of webserver, standalone, pypi and GitHub package.

## 1 Introduction

**Figure 1:**
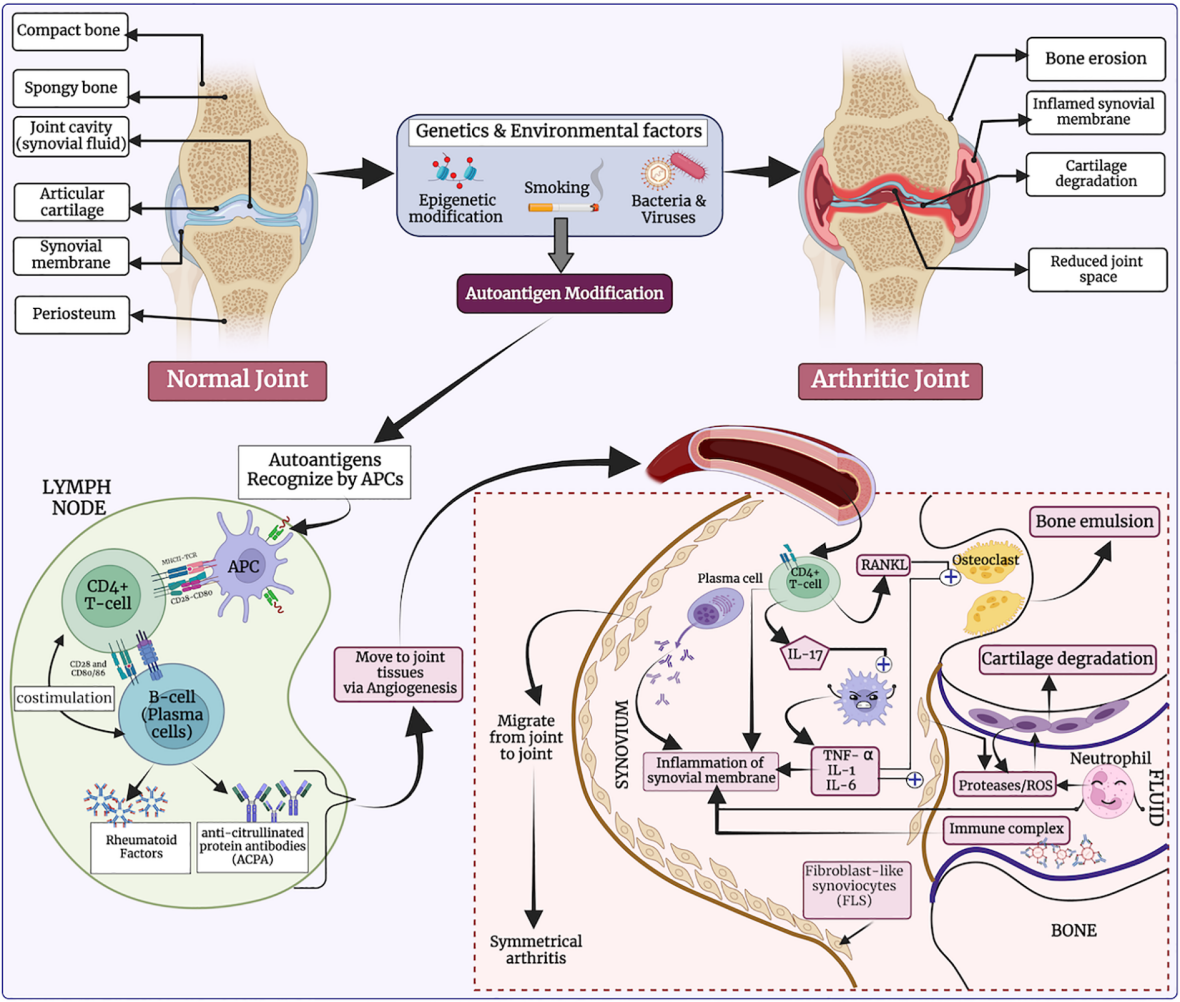
The etiology of rheumatoid arthritis (RA), highlighting how genetic and environmental factors influence T-helper cell activation, ultimately leading to bone erosion and cartilage degradation.

Rheumatoid arthritis (RA) is an incurable, chronic autoimmune joint disorder that exhibits significant clinical heterogeneity (1–3). RA is characterized by abnormal inflammation within the synovial tissue of the joints, which progressively damages both cartilage and bone (4, 5). The global prevalence of RA affects approximately 1% of the world population, translating to millions of individuals (6, 7). Several studies have reported that RA contributes to a diverse range of systemic complications, including cardiovascular disease (5, 8). The pathogenesis of RA is still not fully understood, but it is believed to arise from interactions between genetic predisposition and environmental factors. Several immune cells secrete immune-regulatory molecules that cause inflammation and joint damage—hallmarks of autoimmune diseases (see **Figure 1**) (9). Previously, *in-silico* methods have been developed to predict binders of HLA-DRB1*04:01, as it plays a critical role in RA (10, 11).

In response to genetic and environmental triggers, autoreactive CD4+ T cells become activated and present antigens to B cells, which in turn produce autoantibodies such as rheumatoid factor and anti-citrullinated protein antibodies (12). This autoimmune response is further driven by pro-inflammatory cytokines such as tumor necrosis factor-alpha (TNF-α) and interleukin-6 (IL-6), which promote enhanced immune cell activity and inflammation in the synovium (13). Additionally, macrophages and synovial fibroblasts release chemokines that perpetuate the inflammatory response (14). The dysregulation of the Janus kinase (JAK)/signal transducer and activator of transcription (STAT) signaling pathway is crucial, as it mediates the signaling of several key cytokine receptors involved in RA (2, 15). Together, these pathways contribute to a persistent autoimmune response, leading to chronic joint inflammation and eventual tissue destruction (16).

Traditionally, therapy for RA has primarily focused on disease-modifying anti-rheumatic drugs (DMARDs) (16). These drugs have been reported to reduce pro-inflammatory cytokine production, thereby decreasing the underlying inflammation in the synovium and slowing disease progression (17). Significant progress has been made in the use of DMARDs that target inflammation to prevent joint damage. Methotrexate is considered the first-line therapy due to its proven efficacy and safety (18–20). Additionally, hydroxychloroquine and sulfasalazine are also widely regarded as conventional DMARDs, which can be administered either alone or in combination with methotrexate (21, 22). Apart from DMARDs, glucocorticoids, non-steroidal anti-inflammatory drugs (NSAIDs), and inflammatory cytokine inhibitors (ICIs) are also employed in managing and preventing RA progression (1). However, these traditional drugs have their limitations, including inadequate response, intolerance, high cost, and a number of side effects (23).

In the era of protein therapeutics, one of the key challenges is identifying the antigens or antigenic regions that activate T-helper cells implicated in RA (24). In the past, numerous computational methods have been developed to predict T-helper epitopes responsible for inducing cytokines such as interferon-gamma, TNF-α, IL-4, and IL-5 (25–28). These cytokines play a crucial role in the development of autoimmune diseases like RA. However, to date, there is no in silico computational tool available that predicts T-helper cell-inducing peptides or epitopes that specifically trigger RA.

In this study, we focused on identifying peptides that activate T cells responsible for inducing RA. We extracted 291 experimentally validated MHC class II-binding RA-associated peptides and 165 non-associated peptides from the Immune Epitope Database (IEDB; https://www.iedb.org). To create a robust model, we implemented both alignment-based approaches such as the Basic Local Alignment Search Tool (BLAST) and motif discovery, as well as alignment-free approaches, including machine learning (ML), deep learning (DL), and protein language models (PLMs). In addition, we developed an ensemble model that combines our best performing ML models with motif-based features to achieve higher predictive performance. Finally, we developed a web server and standalone software tool, RAIpred, for predicting, designing, and scanning RA-inducing peptides.

## 2 Materials and methods

### 2.1 Dataset preparation and preprocessing

We gathered experimentally validated data from the IEDB for our study and performed several preprocessing steps to improve the quality of the data used (29). First, we extracted a total of 344 unique RA-inducing peptides as the positive dataset and 176 unique RA non-inducing peptides (not overlapping with the positive dataset) as the negative dataset from IEDB. We observed that RA-associated peptides are binders of both HLA class I and HLA class II molecules (please refer to **Supplementary Figure S1**). Among these, the HLA class I set contained only 46 peptides, while the HLA class II set included 298 peptides. Due to the limited number of HLA class I peptides, we selected only the HLA class II peptides for further analysis.

**Figure 2:**
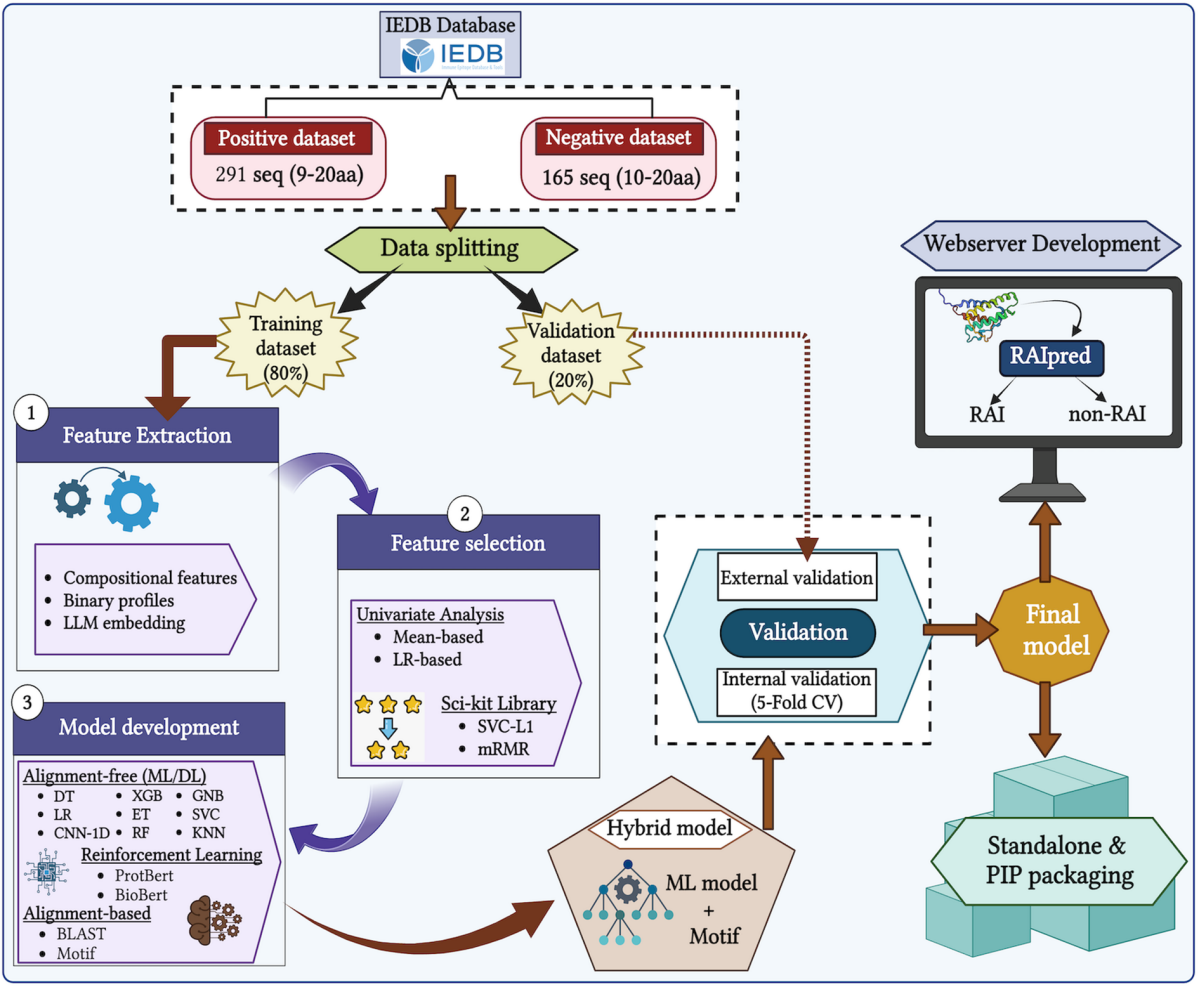
The complete workflow used to develop RAIpred.

Next, we carried out several preprocessing steps, including the removal of duplicate sequences from the negative dataset that overlapped with the positive dataset. We also filtered out sequences from the positive dataset with very low frequency (i.e., those that appeared ≤ 6 times). Furthermore, we retained sequences with lengths between 9 and 20 amino acids. After preprocessing, we were left with 291 sequences in the positive dataset and 165 sequences in the negative dataset. The detailed workflow is shown in **Figure 2**.

### 2.2 Feature generation

Sequence-based features are numerical or categorical representations derived directly from the amino acid sequences of peptides or proteins. These features are essential for computational models such as ML or DL- to understand and make predictions about peptide properties, including immunogenicity, toxicity, antimicrobial activity, or disease association. In the present study, we generated relevant sequence-based features for both RA-inducing and non-inducing peptides. We calculated various composition-based features using the Pfeature software (30). The Pfeature software calculates about 9,189 features using amino acid sequences. We have extracted features such as amino acid composition (AAC), dipeptide composition (DPC), distance distribution of residues (DDR), and many more (please refer to **Supplementary Table S1**).

In addition to composition-based features, we extended feature extraction to include binary profiles. To capture maximum information from the amino acid sequences, we used the Amino Acid Binary Profile (AABP) module of the Pfeature software (30). The generated features served as the basis for implementing ML-based prediction algorithms. Furthermore, we included embeddings from PLMs, specifically those generated using ProtBERT, developed by Rostlab (31). Each feature type carries its own significance and contributes uniquely to the overall prediction performance.

### 2.3 Preliminary analysis

#### 2.3.1 Positional analysis

We created a two-sample logo using the “Two Sample Logo” software to analyze the positional preferences of amino acid residues (32). This method requires input sequences of fixed length. Since the minimum peptide length in our dataset is nine residues, we extracted 9-mers from both the N-terminal and C-terminal of each peptide. These were then concatenated to form a fixed-length sequence of 18 amino acids for each peptide. To generate the two-sample logo plot, these 18-residue sequences from the positive and negative datasets were used as input, enabling the identification of amino acid enrichment or depletion at specific positions between the two classes.

#### 2.3.2 Compositional analysis

To gain deeper insights into the differences in AAC between RA-inducing and non-inducing peptides, we performed compositional analysis on both the positive and negative datasets. For this, we utilized the Pfeature software to compute the AAC of each dataset (30). The AAC is calculated by Pfeature using the following formula;


[1] $$AAC_{i}=\frac{R_{i}}{L}*100$$

Where, AAC_i_ is amino acid composition of residue type i, Ri is the number of residues in i, and L is the length of peptide sequence.

#### 2.3.3 Mean-based univariate analysis

In this analysis, we calculated the absolute mean difference of each feature between the RA-inducing (positive) and non-inducing (negative) classes after normalizing the dataset. To assess the relevance of the generated features, we computed the mean difference for each feature across both groups. Subsequently, we applied an independent t-test to identify the top 5 features with statistically significant *p*-values distinguishing the two classes.

#### 2.3.4 Logistic regression-based analysis

We also employed logistic regression (LR) as a single-feature statistical model to evaluate the relationship between each feature and the target label. We computed the area under the curve (AUC) for each feature to assess its individual relevance in classification.

### 2.4 Alignment-based approach

#### 2.4.1 BLAST search

To annotate peptide sequences, we employed the well-known similarity search tool BLAST (33). Specifically, we used the “blastp-short” algorithm (BLAST+ v2.2.28) designed for short peptide sequences to predict RA-inducing and non-inducing peptides based on sequence similarity.

#### 2.4.2 Motif search

Motifs are short amino acid patterns potentially associated with shared biological functions. This analysis helps identify signature patterns in RA-inducing and non-inducing peptides. These motifs may serve as targets for the development of drugs and therapeutic interventions. We employed the MERCI tool (34), implemented in Perl, to discover motifs exclusive to either the positive or negative dataset using both default and user-defined parameters.

### 2.5 Alignment-free approach

#### 2.5.1 Machine learning models

We used the Scikit-learn Python library to implement various ML algorithms for classification. The classification algorithm consists of decision trees (DT), random forest (RF), multi-layer perceptron (MLP), eXtreme gradient boosting (XGBoost), support vector with the kernel as a radial basis (SVR), ExtraTreesClassifier (ET), LR, k-nearest neighbors (KNN), and Gaussian Naïve Baise (GNB). We employed a GridSearchCV approach to optimize hyperparameters, using AUC as the scoring metric.

#### 2.5.2 Deep learning models

To process sequential data and capture local patterns in peptide sequences, we implemented a one-dimensional convolutional neural network (1D CNN) model for the DL technique. This model is particularly effective in recognizing sequence patterns and dependencies. Hyperparameters were tuned to maximize classification performance for each dataset.

### 2.6 Feature selection

As not all features contribute equally to model performance, we applied two feature selection methods: minimum Redundancy—Maximum Relevance (mRMR) and support vector classifier with L1 regularization (SVC-L1). Feature selection was applied to all composition-based features. The SVC-L1 method selected 34 features, whereas mRMR selected 50 features. We also selected top-performing features based on mean-based univariate and LR analyses.

In addition to this, we have selected top relevant features from mean-based univariate analysis and LR-based analysis. After computing the mean difference among both groups of each feature, we selected 3,782 of 9,189 features. Second, we applied an independent t-test to identify significant features from the stretch of 3,782 features using a *p*-value ≤ 0.05. Finally, we have obtained 305 features with maximum absolute mean difference ranges from 0.001 to 0.14 with significant *p*-values. Upon which, we have deployed ML classifiers over the top 10, 20, 50, 100, 150, 200, 250, and 305 features. Similarly, we have developed ML models on the top 10, 20, and 50 features obtained from LR-based analysis.

### 2.7 Protein language models

Large language models (LLMs), such as PLMs, excel in tasks like peptide classification due to their contextual understanding. We utilized ProtBERT, a pre-trained PLM developed by RostLab, and fine-tuned it to classify RA-inducing and non-inducing peptides. After fine-tuning, the model predicted the class of each input sequence with improved accuracy.

### 2.8 Ensemble method

To develop a more robust classification system, we implemented two ensemble approaches. First, the BLAST-based approach was used to identify disease-causing peptides based on similarity hits, and then the ML approach was used for the prediction of those peptides that are not covered by the BLAST-based approach. Second, the Motif-based approach was used to classify between disease-causing peptides by identifying specific motifs, and then the ML approach was used for the prediction of those peptides not covered by the Motif-based approach.

### 2.9 Model evaluation

To ensure generalizability and prevent overfitting, we followed standard ML practices, including fivefold cross-validation (35, 36). The dataset was split in an 80:20 ratio, with 80% used for training and 20% reserved for external validation. Models were evaluated using both threshold-dependent and threshold-independent metrics:


[2] $$Sensitivity=\frac{TP}{TP+FN}$$

[3] $$Specificity=\frac{TN}{TN+FP}$$

[4] $$Accuracy=\frac{TP+TN}{TP+TN+FP+FN}$$

[5] $$F1-Score=\frac{2TP}{2TP+FP+FN}$$

[6] $$MCC=\frac{\left(TP*TN\right)-\left(FP*FN\right)}{\sqrt{\left(TP+FP\right)\left(TP+FN\right)\left(TN+FP\right)\left(TN+FN\right)}}$$

Where FP is false positive, FN is false negative, TP is true positive and TN is true negative, AUC was used to assess the overall discriminatory power of the models, independent of classification thresholds.

## 3 Results

### 3.1 Preliminary analysis

#### 3.1.1 Positional analysis

**Figure 3:**
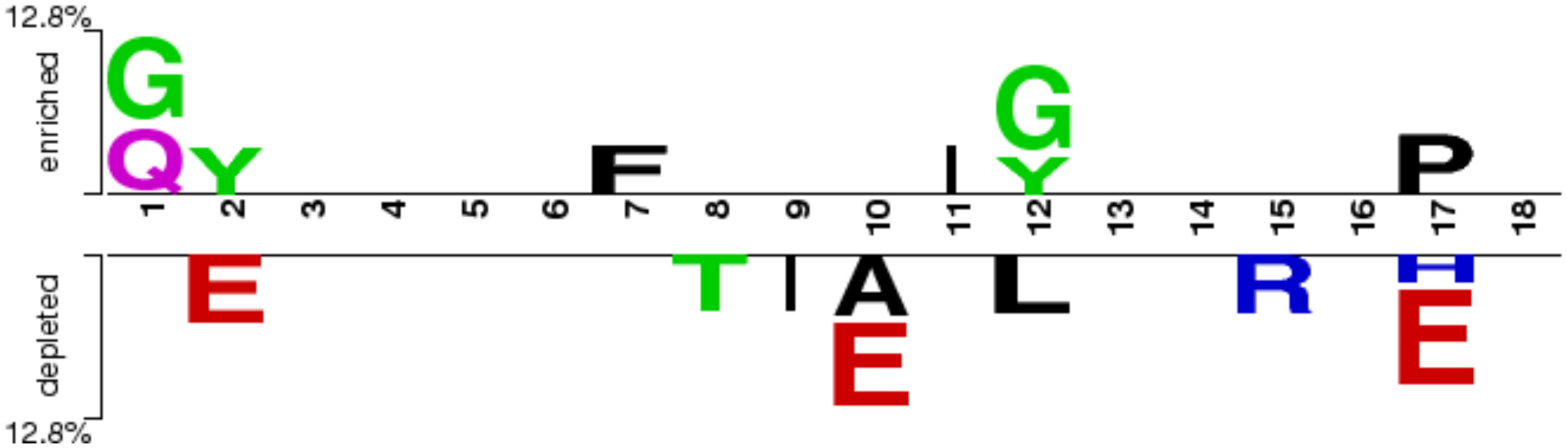
Preference of residues at different positions in RA-inducing and non-inducing peptides.

To determine the most significant positional preferences of amino acid residues within peptides, we employed “Two Sample Logo” for positional analysis. It is important to note that the first nine positions correspond to the N-terminal residues, while the last nine represent the C-terminal residues of the peptides. Our analysis revealed that glycine (G), glutamine (Q), and phenylalanine (F) are predominantly present at the N-terminal of RA-inducing (positive) peptides, whereas isoleucine (I), glycine (G), tyrosine (Y), and proline (P) are enriched at the C-terminal. In contrast, threonine (T) and isoleucine (I) were observed at the N-terminal, and alanine (A), leucine (L), arginine (R), and histidine (H) were commonly found at the C-terminal of non-inducing (negative) peptides. Notably, glutamic acid (E) was consistently prominent across almost all positions in the negative dataset (refer to **Figure 3**).

#### 3.1.2 Compositional analysis

**Figure 4:**
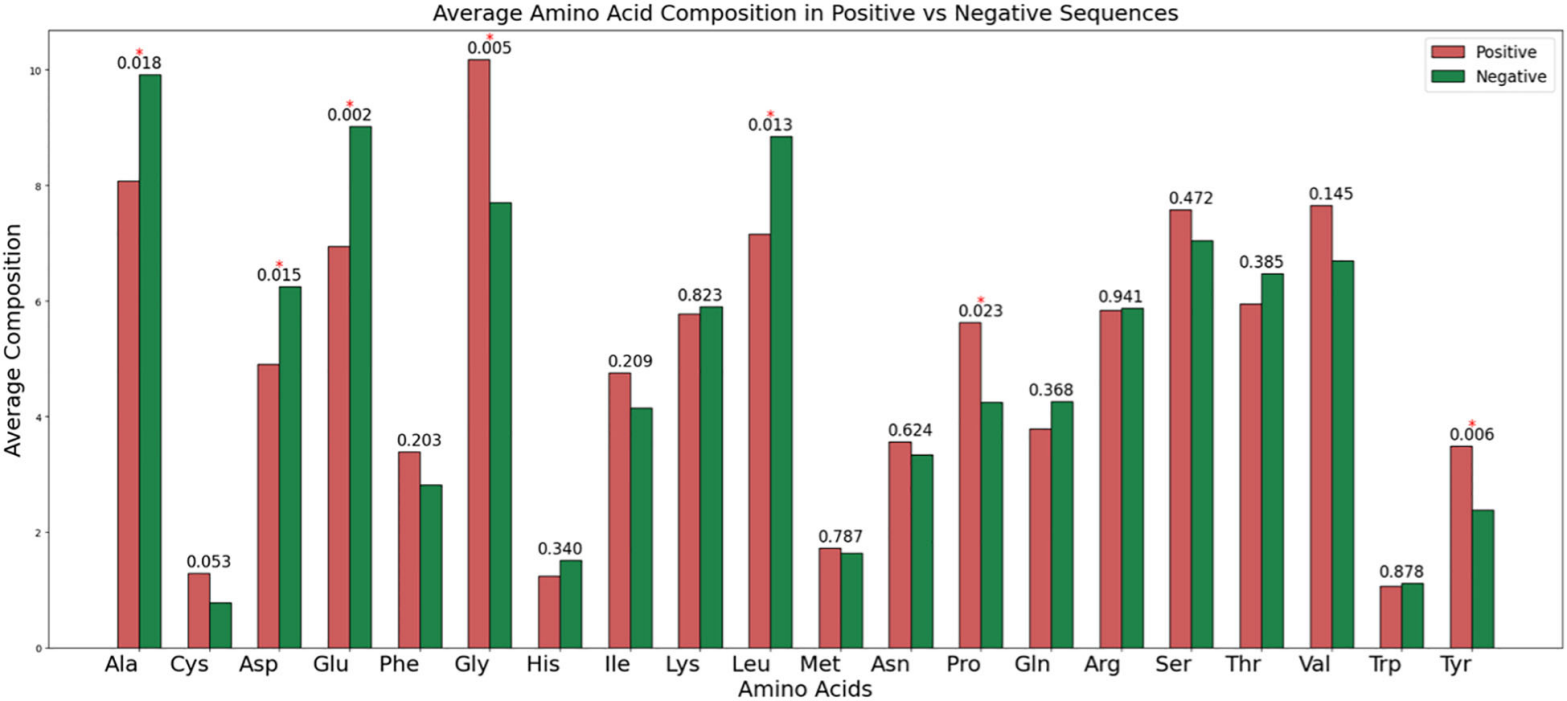
The average amino acid composition among RA-inducing (i.e., positive) and RA non-inducing (i.e., negative) peptides.

We computed the AAC (Using Equation 1) for both RA-inducing (positive) and RA non-inducing (negative) peptides. As shown in **Figure 4**, glycine (G), proline (P), and tyrosine (Y) exhibit the highest average composition in RA-inducing peptides, with statistically significant *p*-values compared to the negative dataset. In contrast, alanine (A), aspartic acid (D), glutamic acid (E), and leucine (L) are significantly more abundant in the RA non-inducing peptides.

#### 3.1.3 Mean-based univariate analysis

We finally selected 305 features based on their higher mean differences between the positive and negative datasets, along with statistically significant *p*-values. As shown in **Table 1**, the composition-enhanced transition and distribution (CeTD) features such as CeTD_21_HB and CeTD_25_p_VW3 exhibited the highest mean differences of 0.084 and −0.140, respectively. For the complete list of selected features and their rankings, please refer to **Supplementary Table S2**.

**Table 1:** Significantly preferred, or not preferred, features in RA-inducing peptides in terms of difference in mean values between RA-inducing and non-inducing peptides.

Feature	Mean value		Difference in mean (Inducing - non-inducing)
	RA-inducing	Non-inducing	
Features preferred in RA-inducing peptides			
CeTD_21_HB	0.466	0.382	0.084
SER_Y	0.275	0.196	0.079
PRI_NE	0.340	0.265	0.075
PRI_NE_pH	0.340	0.265	0.075
CeTD_11_SS	0.373	0.299	0.074
Features not preferred in RA-inducing peptides			
CeTD_25_p_VW3	0.306	0.445	−0.140
CeTD_100_p_VW3	0.320	0.453	−0.133
SER_E	0.382	0.514	−0.132
CeTD_75_p_VW3	0.340	0.471	−0.131
CeTD_50_p_VW3	0.330	0.458	−0.128

#### 3.1.4 Logistic Regression-based analysis

To identify the best features based on their individual performance, we also applied an LR classifier on 9,189 features calculated using the Pfeature tool. We observed that the top AUC features belong to the Bond Composition (BTC) and CeTD feature categories. The features named BTC_T, BTC_S, and BTC_H achieved a maximum AUC of 0.69, while features CeTD_75_p_VW3, CeTD_100_p_VW3, and CeTD_50_p_VW3 achieved an AUC of 0.68. The top 10 features with their performance are shown in **Table 2**, and detailed results are provided in **Supplementary Table S3**.

**Table 2:** The performance of single feature based LR models developed using the top ten features.

Feature name	Sensitivity	Specificity	Accuracy	AUC	Kappa	MCC
BTC_T	65.64	66.06	65.79	0.69	0.30	0.31
BTC_S	66.32	67.88	66.89	0.69	0.32	0.33
BTC_H	65.98	67.27	66.45	0.69	0.31	0.32
CeTD_75_p_VW3	56.70	66.06	60.09	0.68	0.21	0.22
CeTD_100_p_VW3	56.70	66.06	60.09	0.68	0.21	0.22
CeTD_50_p_VW3	68.73	53.33	63.16	0.68	0.22	0.22
CeTD_100_p_HB3	71.13	53.33	64.69	0.66	0.24	0.24
CeTD_75_p_HB3	71.13	53.33	64.69	0.66	0.24	0.24
CeTD_50_p_HB1	62.89	63.03	62.94	0.66	0.24	0.25
CeTD_50_p_SS3	62.89	63.03	62.94	0.66	0.24	0.25

### 3.2 Alignment-based approach

We used both alignment-free (ML techniques) and alignment-based (motif & BLAST) methods, as explained in previous sections. Each strategy has its own advantages and limitations. Alignment-based techniques generally have low sensitivity but high specificity, as their performance depends on the presence of motifs or sequence similarity. In contrast, alignment-free ML-based approaches are more generalizable and not dependent on sequence similarity. We developed ensemble or hybrid approaches combining BLAST and Motif to leverage the strengths of both methods.

#### 3.2.1 BLAST

In the BLAST-based approach, we first prepared a BLAST-formatted database using the training dataset. Then, we searched the query sequences (from the validation dataset) against the training database to find hits at various e-values ranging from 1e-5 to 1e+3. A query sequence was categorized as positive if the top hit was positive and negative if the top hit was negative. The detailed BLAST results on the validation data are shown in **Table 3**.

**Table 3:** The performance of the BLAST method in terms of coverage of RA-inducing and non-inducing peptides at different e-values.

BLAST + CeTD (validation dataset)					
E-value	Number of hits	RA-inducers	RA non-inducers	Correct pos	Correct neg
1.00E+10	92	46	46	10	23
1.00E+03	92	46	46	10	23
1.00E+02	87	44	43	9	20
1.00E+01	66	32	34	6	13
1.00E+00	53	28	25	6	12
1.00E-01	47	25	22	20	10
1.00E-02	39	21	18	4	10
1.00E-03	30	16	14	4	9
1.00E-04	20	12	8	4	6
1.00E-05	14	7	7	2	5
1.00E-08	2	1	1	0	1

Correct pos, correct positive; Correct neg, correct negative.

Next, we combined the predicted labels obtained using CeTD features with BLAST scores to improve the performance of our XGB models. We attained a maximum AUC of 0.77 on the validation dataset at an e-value of 1.00E+01. However, such a high e-value could indicate a random chance of getting hits. The complete result table is provided in **Supplementary Table S4**. To develop a more robust model, we further explored a motif-based approach.

#### 3.2.2 Motif

In the motif-based approach, we identified K exclusive motifs using the MERCI tool. MERCI provides different parameters to generate specific motifs based on positive and negative datasets. We calculated exclusive motifs using the “None,” KOOLMAN-ROHM, and BETTS-RUSSELL classification methods, assigning a score of +0.5 if the motif was found in a positive sequence and 0 if no match was found. We then combined the predicted labels from the best model with motif scores. The best performing motifs were obtained using the BETTS-RUSSELL classification method. The detailed list of motifs with their occurrence in the RA-inducing dataset is provided in **Table 4**.

**Table 4:** List of highly abundant motifs in RA-inducing peptides.

Motif	Coverage in RA inducers
tiny polar hydrophobic G hydrophobic	22
hydrophobic L hydrophobic aliphatic small	20
hydrophobic polar hydrophobic tiny polar hydrophobic hydrophobic polar	19
aliphatic aliphatic hydrophobic polar polar aliphatic	19
small S hydrophobic G	18
hydrophobic polar A G hydrophobic	17
G small small G small	17
P hydrophobic polar polar hydrophobic	17
tiny hydrophobic S hydrophobic hydrophobic hydrophobic	16
tiny S hydrophobic G	15
tiny S tiny tiny	15

Polar, H,K,R,D,E,Y,W,T,C,S,N,Q; charged, D,E,R,H,K; negative, D,E; positive, R,H,K; small, A,G,C,S,P,N,D,T,V; tiny, A,G,C,S; hydrophobic, H,F,W,Y,I,L,V,M,K,T,A,G,C; aromatic, H,F,W,Y; aliphatic, I,L,V.

### 3.3 Alignment free approach

In this study, multiple classifiers were employed to differentiate between RA-inducing and non-inducing peptides. The predictive performance of these models was systematically assessed using standard evaluation metrics, with the corresponding calculations derived from the formulas provided in Equations 2–6. This ensured a comprehensive and rigorous assessment of classification accuracy, reliability, and generalizability.

#### 3.3.1 Machine learning-based analysis

We applied multiple ML-based classifiers to composition-based features and AABP features. Results are shown in **Supplementary Table S5**. Our findings demonstrate that among the various composition-based features, the CeTD features performed exceptionally well. Using CeTD based features, we achieved a maximum accuracy and AUC of 71% and 0.75 on the training dataset and 66.30% and 0.75 on the validation dataset, with balanced sensitivity and specificity using the XGB classifier. **Table 5** shows the performance of the best model across all composition and binary profile-based features on the validation dataset.

**Table 5:** The performance of the ML models using the best set of features on the validation dataset.

Feature name	ML model	Sensitivity	Specificity	Accuracy	AUC	Kappa	MCC
CeTD	XGB	61.02	75.76	66.30	0.75	0.33	0.35
TPC	ET	62.71	69.70	65.22	0.74	0.30	0.31
ALLCOMP	LR	57.63	75.76	64.13	0.73	0.30	0.32
APAAC	ET	61.02	63.64	61.96	0.72	0.23	0.24
DPC	KNN	61.02	69.70	64.13	0.71	0.28	0.30
AAC	ET	59.32	60.61	59.78	0.70	0.19	0.19
BTC	GNB	50.85	75.76	59.78	0.69	0.23	0.26
DDR	ET	52.54	75.76	60.87	0.67	0.25	0.28
CTC	SVC	62.71	66.67	64.13	0.65	0.27	0.28
PRI	LR	55.93	69.70	60.87	0.63	0.23	0.25
AABP	KNN	55.93	51.52	54.35	0.59	0.07	0.07

XGB, extreme gradient boosting; ET, extra tree; LR, logistic regression; KNN, k-nearest neighbors; GNB, Gaussian Naïve Baise; SVC, support vector classifier; AUC, area under curve; kappa, Cohen’s kappa coefficient; MCC, Mathew’s correlation coefficient.

#### 3.3.2 Deep learning-based analysis

We applied a 1D-CNN on various composition-based features as well as on amino acid binary profile-based features. The 1D-CNN performed well on tri-peptide composition features, achieving an AUC of 0.69 on the validation dataset. Detailed results of the 1D-CNN model on different features are provided in **Supplementary Table S6**. Performance summary is shown in **Table 6**.

**Table 6:** The performance of the 1D-CNN model over different types of features on the validation dataset.

Feature name	Sensitivity	Specificity	Accuracy	AUC	Kappa	MCC
AAC	88.14	21.21	64.13	0.60	0.11	0.13
DPC	50.85	63.64	55.44	0.67	0.13	0.14
TPC	55.93	72.73	61.96	0.69	0.26	0.28
BTC	0.00	100.00	35.87	0.38	0.00	0.00
DDR	55.93	45.46	52.17	0.54	0.01	0.01
CTC	52.54	66.67	57.61	0.60	0.17	0.19
PRI	45.76	69.70	54.35	0.64	0.14	0.15
CeTD	71.19	48.49	63.04	0.68	0.20	0.20
APAAC	59.32	51.52	56.52	0.65	0.10	0.11
AAB	55.93	54.55	55.44	0.60	0.10	0.10
ALLCOMP	57.63	69.70	61.96	0.72	0.25	0.26

AUC, area under curve; kappa, Cohen’s kappa coefficient; MCC, Mathew’s correlation coefficient.

#### 3.3.3 Feature selection techniques

To select the most relevant features, we applied two feature selection techniques—SVC-L1 and mRMR. Using SVC-L1, we selected 34 composition-based features. As shown in **Table 7**, we achieved a maximum AUC of 0.72 on the validation dataset using the SVC.

**Table 7:** The performance of the ML models developed using SVC-L1–selected features on the validation dataset.

Model	Sensitivity	Specificity	Accuracy	AUC	Kappa	MCC
DT	62.71	48.49	57.61	0.58	0.11	0.11
RF	59.32	72.73	64.13	0.70	0.29	0.31
LR	59.32	66.67	61.96	0.71	0.24	0.25
XGB	66.10	69.70	67.39	0.69	0.34	0.34
KNN	61.02	69.70	64.13	0.71	0.28	0.30
GNB	62.71	66.67	64.13	0.68	0.27	0.28
ET	69.49	54.55	64.13	0.69	0.24	0.24
SVC	62.71	75.76	67.39	0.72	0.35	0.37
MLP	62.71	66.67	64.13	0.71	0.27	0.28

DT, decision tree; RF, random forest; LR, logistic regression; XGB, extreme gradient boosting; KNN, k-nearest neighbors; GNB, Gaussian Naïve Baise; ET, extra tree; SVC, support vector classifier; MLP, multilayer perceptron; AUC, area under curve; kappa: Cohen’s kappa coefficient; MCC, Mathew’s correlation coefficient.

We also applied the mRMR technique, which selected 50 composition-based features and achieved a maximum AUC of 0.73 using the RF classifier. These results are presented in **Table 8**.

**Table 8:** The performance of the ML models developed using mRMR-selected features on the validation dataset.

Model	Sensitivity	Specificity	Accuracy	AUC	Kappa	MCC
DT	55.93	66.67	59.78	0.65	0.21	0.22
RF	59.32	69.70	63.04	0.73	0.27	0.28
LR	66.10	75.76	69.57	0.72	0.39	0.40
XGB	55.93	75.76	63.04	0.71	0.28	0.31
KN	57.63	72.73	63.04	0.69	0.27	0.29
GNB	79.66	39.39	65.22	0.60	0.20	0.21
ET	52.54	72.73	59.78	0.74	0.22	0.24
SVC	66.10	69.70	67.39	0.71	0.34	0.34
MLP	64.41	66.67	65.22	0.67	0.29	0.30

DT, decision tree; RF, random forest; LR, logistic regression; XGB, extreme gradient boosting; KNN, k-nearest neighbors; GNB, Gaussian Naïve Baise; ET, extra tree; SVC, support vector classifier; MLP, multilayer perceptron; AUC, area under curve; kappa, Cohen’s kappa coefficient; MCC, Mathew’s correlation coefficient.

Additionally, we implemented mean-based univariate analysis and LR-based feature selection. We applied ML techniques to 305 features, as well as to the top 200, 150, 100, 50, 20, and 10 selected features. Using this approach, we achieved the highest AUC of 0.73 for the top 100, 150, and 200 features. Results are presented in **Table 9**. For detailed results, refer to **Supplementary Table S7**. Performance of various ML algorithms on the top 10, 20, and 50 features selected through LR is provided in **Supplementary Table S8**.

**Table 9:** The performance of the best ML models developed using different sets of top features on the validation dataset. Features were selected using mean-based univariate analysis.

Total feature	ML model	Sensitivity	Specificity	Accuracy	AUC	Kappa	MCC
Top 305	RF	57.63	66.67	60.87	0.72	0.22	0.23
Top 200	RF	66.10	69.7	67.39	0.73	0.34	0.34
Top 150	RF	59.32	72.73	64.13	0.73	0.29	0.31
Top 100	ET	67.80	66.67	67.39	0.73	0.33	0.33
Top 50	ET	61.02	66.67	63.04	0.70	0.26	0.27
Top 20	SVC	50.85	75.76	59.78	0.70	0.23	0.26
Top 10	SVC	59.32	69.70	63.04	0.69	0.27	0.28

RF, random forest; ET, extra tree; SVC, support vector classifier.

### 3.4 Protein language model-based analysis

We used two pre-trained large language models—protBERT and BioBERT—for this study. Each model was fine-tuned by changing the number of epochs, which allowed the model to learn and update its parameters depending on the sequences it processed. An epoch is defined as one complete pass through the whole training dataset. The best results were obtained at epoch 10 for protBERT and epoch 3 for BioBERT, achieving maximum AUCs of 0.71 and 0.67, respectively, on the fine-tuned models. Results are shown in **Table 10**.

**Table 10:** Best performance of PLM models on the validation dataset, models were fine-tuned on the training dataset.

Model name	Sensitivity	Specificity	Accuracy	AUC	MCC
ProtBERT	0.81	0.58	0.73	0.71	0.40
BioBERT	0.69	0.58	0.65	0.67	0.26

Furthermore, we extracted embeddings from the fine-tuned models and applied various ML algorithms. However, these models did not perform well on our dataset. Results for PLM-based models are presented in **Supplementary Table S9**.

### 3.5 Ensemble model

An ensemble model was developed by combining the best-performing ML model (based on CeTD features) with the motif-based approach. BETTS-RUSSELL classification in MERCI (parameters: fp = 2, fn = 0, g = 0, k = 20) identified the best motifs that covered the maximum validation dataset. We combined these motif scores with the best ML classifier. As shown in **Table 11**, this ensemble model achieved the highest AUC of 0.80 and MCC of 0.45 on the validation dataset. These exclusive motifs help in identifying specific regions in proteins that may cause RA. This ensemble model is also implemented in the prediction module of the RAIpred web server for ease of access.

**Table 11:** The performance of the hybrid model developed using exclusive positive motifs (with MERCI classification – None, KOOLMAN-ROHM and BETTS-RUSSELL).

Classification method	Requested number of motif	Sensitivity	Specificity	Accuracy	AUC	Kappa	MCC
None	K20	67.80	66.67	67.39	0.70	0.33	0.33
KOOLMAN-ROHM	K20	71.19	66.67	69.57	0.75	0.36	0.37
BETTS-RUSSELL	K20	71.19	75.76	72.83	0.80	0.44	0.45

K, number of requested motifs; AUC, area under curve; kappa, Cohen’s kappa coefficient; MCC, Mathew’s correlation coefficient.

### 3.6 Web server design

We developed “RAIpred,” available at https://webs.iiitd.edu.in/raghava/raipred/, to provide a user-friendly web interface for predicting HLA class II binding RA-inducing or non-inducing peptides only. The platform includes “Prediction,” “Design,” “Protein Scan,” and “Motif Scan” modules. The Protein Scan module identifies RA-inducing regions in a given protein sequence. The design module enables generation of all possible analogs of a given peptide and evaluates their RA-inducing potential. The Protein Scan module identifies RA-inducing regions in the protein. The Motif Scan module uses MERCI to map RA-inducing motifs in the query sequence. The platform is responsive and accessible on desktops, laptops, and smartphones.

We also developed a standalone Python tool called “RAIpred” to assist in identifying potentially disease-causing regions in peptides or proteins. The tool can be downloaded from the web server’s download page. The web server is powered by HTML5, Java, CSS3, and PHP and supports a variety of devices, including desktop, tablet, mobile, and iMac.

## 4 Discussion

RA is caused by gradual loss of self-tolerance in genetically vulnerable individuals due to various environmental stressors. Both the genetic and environment factors are significantly responsible for the onset of the disease. The ability of peptides or proteins to selectively bind arthritogenic amino acid sequences for presentation to auto-reactive T lymphocytes is provided by a common epitope in the peptide-binding groove area of MHC class II molecules (37). These T cells generate inflammatory responses by releasing excessive amounts of cytokines and by activating B cells, which are further responsible for the excessive production of autoantibodies and lead to destruction and bone erosion. As these arthritogenic peptides are responsible for inducing T-cell response, they can be targeted for therapeutic purposes by modifying their properties using peptide cyclization, chemical modification, or other *in-vitro* approaches.

Few ML-based techniques have been developed in the past to combat RA. One of these techniques, was developed in order to forecast how well biologic drugs will work in treating patients with RA and AS (ankylosing spondylitis), with an AUC of 0.64 based on the validation dataset. Furthermore, it demonstrates that the most significant predictors of therapy responses were patient self-reporting scales, the Bath Ankylosing Spondylitis Functional Index (BASFI) in AS patients, and the patient global assessment of disease activity (PtGA) in RA patients (38). Another study uses ML to predict patient relapses based on blood test results and ultrasound examination data (39). Prasad et al. developed ATRPred, an ML-based technique that uses clinical and demographic characteristics to predict how well RA patients will respond to anti-TNF treatment (40). One study uses genetic information from SNPs in non-HLA genes to predict RA (41). To the best of our knowledge, no method has yet been developed to anticipate the T-helper cell-inducing peptides or epitopes that trigger RA.

In this study, we have made a systematic approach for the classification of RA-inducing peptides. We have extracted 291 RA-inducing peptides and 165 RA non-inducing peptides from the IEDB. In the preceding sections, we observed a few key insights from the comprehensive analysis of RA-inducing and non-inducing peptides. Here, compositional analysis indicates that RA-inducing peptides have the highest average composition of glycine and proline as compared to non-inducing peptides, which might be responsible for peptide binding to MHC (42). In addition to this, positional analysis further marks distinct amino acid preferences for the N-terminal and C-terminal regions in positive and negative datasets, emphasizing the differential roles of residues such as glycine, threonine, and alanine.

In classification modelling, both alignment-based (BLAST and Motif) and alignment-free methods were implemented. BLAST-based approach demonstrated slightly increased performance at higher e-values, which depict the random chances of getting hits. While the Motif-based approach gave the highest number of correct hits for the validation dataset. In the present study, among different composition-based features, we have observed that CeTD composition-based features outperformed all. We have reported maximum accuracy and AUC over the training dataset as 71% & 0.75 and 66.30% & 0.75 over validation dataset with balanced sensitivity and specificity by applying the XGB classifier. This result underscores that CeTD features capture physicochemical peptide properties for our dataset, which is critical for accurate predictions. Finally, we have combined motif analysis and an ML-based methodology to develop an ensemble method. On the validation dataset, an ensemble-based method gets a maximum AUC of 0.80 and an MCC of 0.45. All the performance metrices calculated using Equations 2–6. The integration of compositional insights and ML algorithms enabled the development of a robust tool “RAIPred,” for HLA class II binding RA-inducing peptide prediction. In order to provide the scientific community an easy and user-friendly approach for the prediction of RA-inducing peptides, we have developed RAIpred as a web server (https://webs.iiitd.edu.in/raghava/raipred/), a standalone package (https://webs.iiitd.edu.in/raghava/raipred/download.html) and it is also available on Github (https://github.com/raghava/raipred) and python package (https://pypi.org/project/raipred/).

### 4.1 Applications of RAIpred

- Assess the HLA class II binding RA-inducing potential of novel peptides/proteins before therapeutic or GMO applications.
- Design therapeutic peptides with optimized physicochemical properties and screen them as HLA class II binding RA-inducers or non-inducers.
- Map antigenic epitopes responsible for RA using the Protein Scan module.
- Design peptide analogs with single-residue modifications using the Design Module to evaluate HLA class II binding RA-inducing potential.

## 5 Conclusion

Peptide-based therapeutics are increasingly popular due to their target specificity and clinical success. Risk assessment of these proteins is essential for preventing them from causing some severe side-effects or being involved in disease development. There are several peptide-based drugs approved by the FDA for the treatment of RA and other autoimmune disorders. RAIpred is a reliable and accurate tool for identifying HLA class II binding RA-inducing peptides, aiding in the development of targeted therapeutics. It also provides deep insights into peptide functionality and helps discover novel bioactive peptides with pharmaceutical relevance.

## Data Availability

The dataset used in this study is publicly available for download at https://webs.iiitd.edu.in/raghava/raipred/download.html. Further inquiries can bedirected to the corresponding author.
