# Supplementary figures and images for: In-silico tool for predicting and scanning rheumatoid arthritis-inducing peptides in an antigen
Source: Front Immunol. 2025 Sep 1;16:1630863. doi: 10.3389/fimmu.2025.1630863 (PMC12433991; doi:10.3389/fimmu.2025.1630863)

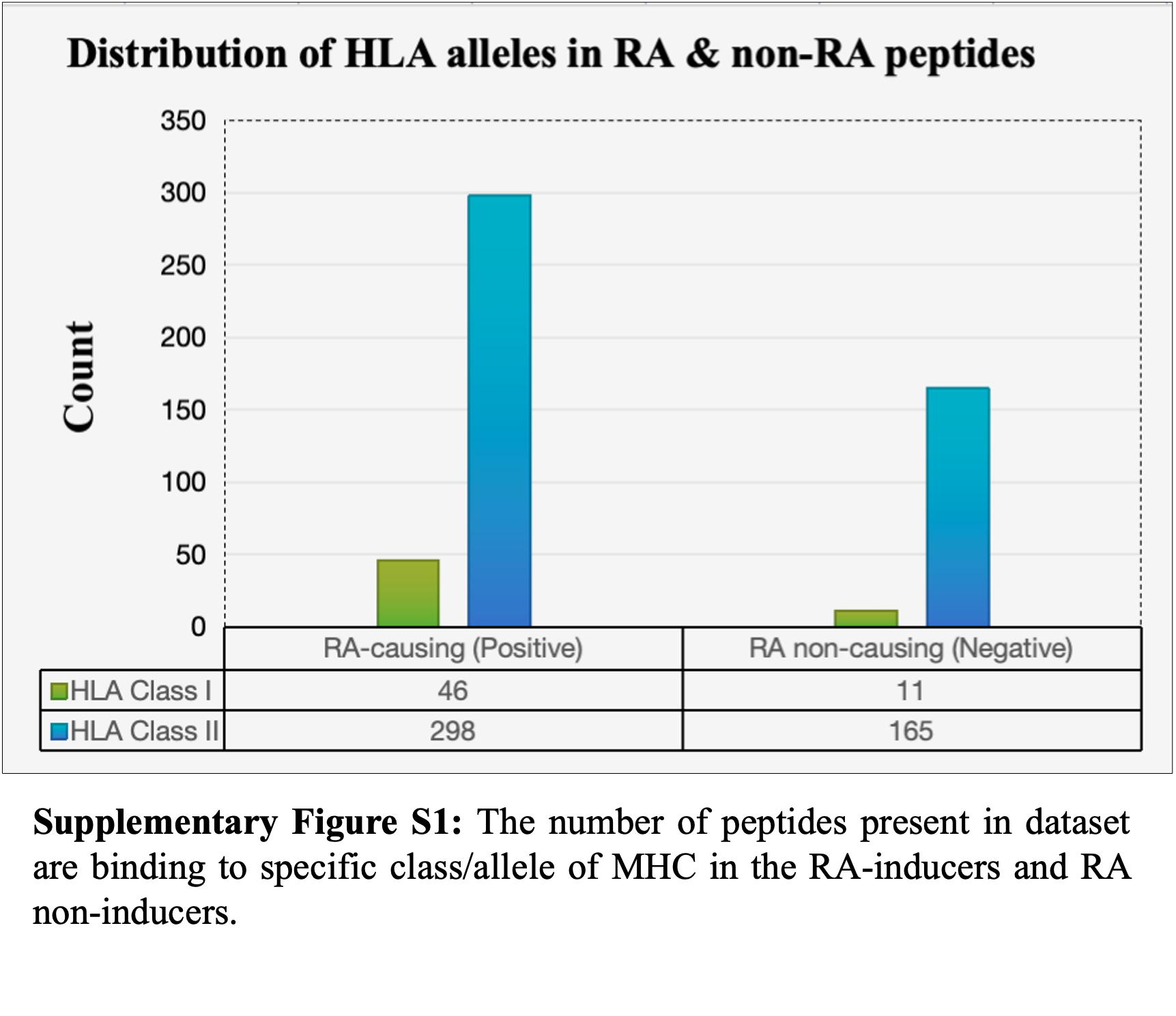

Supplement: Supplementary file 2 [file Image1.png]
